# Compensatory effect of fibrinogen in a patient with bone marrow aplasia, septic shock, and severe thrombocytopenia guided by thromboelastometry: a case report

**DOI:** 10.5935/0103-507X.20180056

**Published:** 2018

**Authors:** Tomaz Crochemore, Felicio Aragão Savioli, Camila Menezes Pessoa, Adriana Abreu Resende, Roberto Camargo Narciso

**Affiliations:** 1 Unidade de Terapia Intensiva, Hospital Leforte Morumbi - São Paulo (SP), Brasil.

**Keywords:** Thromboelastography, Thrombocytopenia, Fibrinogen, Septic shock, Bone marrow diseases, Bone marrow/abnormalities

## Abstract

Platelet transfusion is a common practice to prevent spontaneous bleeding or bleeding due to invasive procedures. Transfusion of allogeneic blood components is associated with increased mortality and a worse clinical outcome. The clot strength is assessed by thromboelastometry and determined by the interaction between platelets and fibrinogen. The compensatory effect of high levels of fibrinogen on clot strength in patients with thrombocytopenia has been demonstrated in different clinical settings including sepsis. We report the case of a patient with severe thrombocytopenia whose thromboelastometry showed clot strength that was compensated for by the increase in plasma fibrinogen levels as an acute phase reactant of septic patients. Here, we report a case of a 62-year-old female diagnosed with bone marrow aplasia admitted in the intensive care unit with septic shock and severe thrombocytopenia. During the first 24 hours in the intensive care unit, she presented acute respiratory insufficiency and circulatory shock. The use of invasive mechanical ventilation and norepinephrine was required. Her chest X-ray showed bilateral lung injury. Thus, bronchoscopy with bronchoalveolar lavage was requested. Thromboelastometry was performed and resulted in a normal coagulable profile. Despite severe thrombocytopenia (1,000/mm^3)^, fibrinogen levels were increased (1,050mg/dL) due to septic shock. Bronchoscopy was performed without any active or further bleeding. Here, we report the use of thromboelastometry in the diagnosis of coagulation disorders, preventing unnecessary prophylactic platelet transfusion.

## Abstract (pt)

A transfusão de concentrado de plaquetas é prática comum para prevenção de sangramento espontâneo ou decorrente de procedimentos invasivos; sabe-se que a transfusão de componentes alogênicos do sangue se associa a aumento da mortalidade e piora do desfecho clínico. A força do coágulo é avaliada por meio da tromboelastometria rotacional e determinada pela interação entre plaquetas e fibrinogênio. O efeito compensatório do incremento na concentração sérica de fibrinogênio na força do coágulo, em pacientes com trombocitopenia, tem sido demonstrado em diferentes contextos clínicos, incluindo sepse. Relatamos o caso de uma paciente com trombocitopenia grave, cujo resultado da tromboelastometria rotacional demonstrou efeito compensatório na força do coágulo determinada pelos níveis plasmáticos aumentados de fibrinogênio como reagente de fase aguda em pacientes sépticos. Relatamos o caso de uma paciente de 62 anos com diagnóstico de aplasia de medula óssea admitida a uma unidade de terapia intensiva com choque séptico e trombocitopenia grave. Nas primeiras 24 horas na unidade de terapia intensiva, ela apresentou quadro clínico de insuficiência respiratória aguda e choque. Foi necessário utilizar ventilação mecânica invasiva e fármaco vasoativo. A radiografia de tórax mostrou padrão de lesão pulmonar bilateral. Desta forma, foi solicitada broncoscopia com lavagem broncoalveolar para investigação diagnóstica. Conduziu-se uma tromboelastometria rotacional, e seu resultado mostrou perfil de coagulação normal. Apesar da trombocitopenia grave (1.000/mm^3)^, os níveis de fibrinogênio aumentaram (1.050mg/dL) devido ao choque séptico. A broncoscopia foi realizada sem que subsequentemente ocorresse sangramento ativo. Este caso relata o uso da tromboelastometria como ferramenta diagnóstica em distúrbios da coagulação de pacientes graves, permitindo prevenir o uso desnecessário de transfusões profiláticas de concentrado de plaquetas.

## INTRODUCTION

The cell-based model of coagulation described in 2001 by Hoffman et al., demonstrated the importance of the membrane surface of cells for thrombin generation and clot formation, whose initial trigger is determined by the tissue factor released by the endothelium. The process of clot formation is composed of four consecutive phases: initiation, amplification, propagation and stabilization of the clot. The extrinsic and intrinsic pathway of coagulation function in a dependent and sequential way in the initial phases for the production of thrombin.^(1)^

Conventional coagulation tests such as activated partial thromboplastin time or prothrombin time are weak predictors of bleeding in critically ill patients.^(2)^ Conventional coagulation tests fail to identify hypercoagulability and hyperfibrinolysis, accessing only 5% of the thrombin generation.^(2,3)^

Viscoelastic tests allow for early detection of coagulopathy and can predict massive transfusion. Viscoelastic tests can also guide goal-directed therapy with specific hemostatic drugs, coagulation factor concentrates, and allogeneic blood products.^(4,5)^

Fibrinogen is an acute phase protein that is synthesized in the liver in response to inflammatory signals. The concentration of fibrinogen increases with inflammation including sepsis.^(6)^ Both the fibrinogen levels and the platelet count are determinants of clot strength as shown by the maximum clot firmness (MCF), which is a parameter of rotational thromboelastometry.^(7,8)^

We report a clinical case of a septic patient marked by severe thrombocytopenia owing to bone marrow aplasia. Bronchoscopy was required due to acute respiratory impairment, and rotational thromboelastometry was performed to guide the transfusion and maintain safety during the procedure. No local or distant bleeding was observed in the patient.

## CASE REPORT

**Figure 1:**
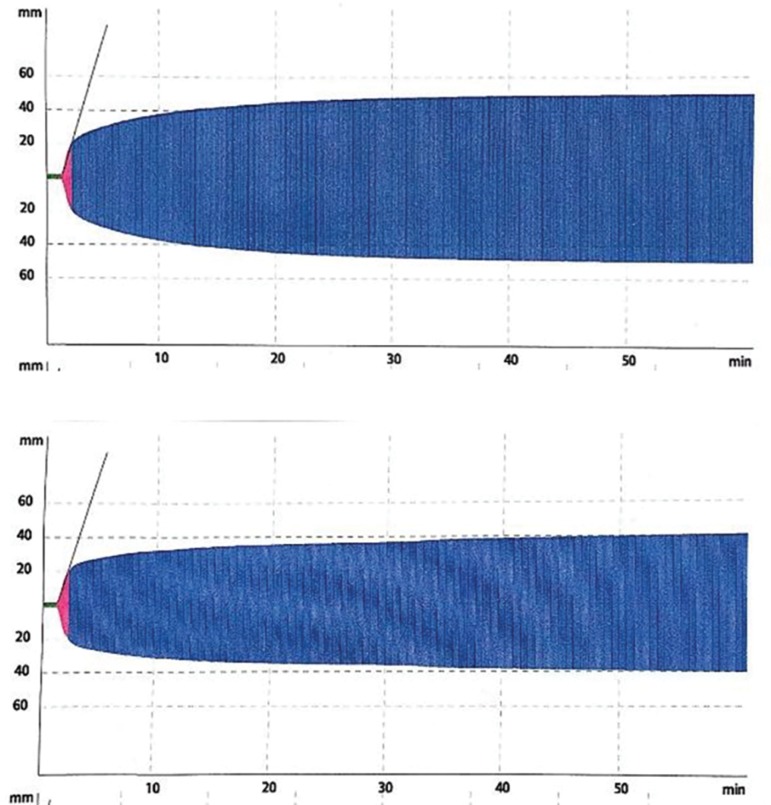
Results of thromboelastometry.

**Figure 2:**
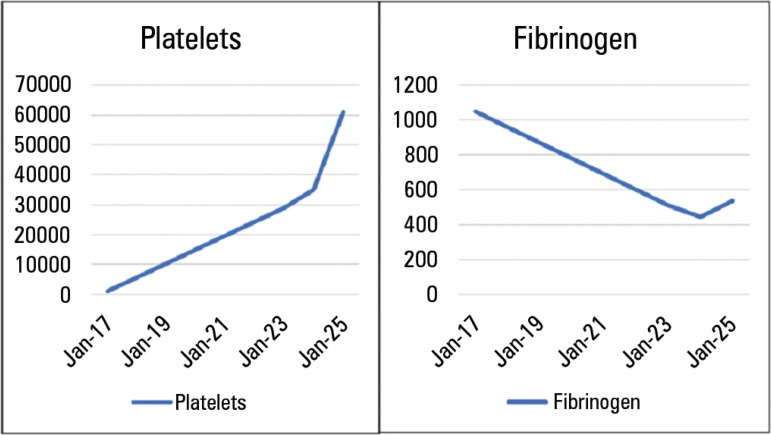
Evolution of platelet count and fibrinogen concentration.

A 62-year-old female, with primary bone marrow aplasia was admitted in the intensive care unit (ICU) with septic shock, hematomas and petechiae spread throughout the body. A physical examination revealed impaired conscious level, tachycardia, and hypotension. Laboratory examination revealed the following: hemoglobin 8.2g/dL, leukocytes 290/mm^3^, platelets 1000/mm^3^, fibrinogen 1050mg/dL, international normalized ratio 1.1, C-reactive protein 52mg/dL, and creatinine 1.1mg/dL (Table 1). Orotracheal intubation was performed due to respiratory insufficiency and an impaired conscious level. Norepinephrine and antibiotics were started. A computed tomography was performed showing bilateral alveolar infiltrate. Bronchoscopy and bronchoalveolar lavage were requested to investigate the etiological cause. Due to severe thrombocytopenia, thromboelastometry was requested to determine whether the bronchoscopy could be performed safely. EXTEM (Extrinsic rotational thromboelastometry) showed MCF of 50 millimeters (mm), ML (Maximum Lysis) of 0%, and FIBTEM (Fibrinogen rotational thromboelastomery) showed MCF of 40mm (Figure 1 and Table 2). The patient presented with a normal coagulable profile according to thromboelastometry even with extremely low platelet quantitative levels (1000/mm^3)^. Bronchoscopy was safely performed with signs of bilateral alveolar hemorrhage, with the presence of organized clots in the inferior lobe segment but without active bleeding. The patient was extubated seven days after bronchoscopy, without any signs of bleeding. Laboratory test results showed an increase in platelet counts as well as a reduction in fibrinogen concentration with the improvement of sepsis (Figure 2). She was discharged from the ICU three days after extubation.

**Table 1:** Laboratory results

Laboratory tests	Results	Reference range
Hemoglobin	8.2g/dL	12 - 16g/dL
Leukocytes	290/mm^3^	4,000 - 11,000/mm^3^
Platelet count	1,000/mm^3^	150,000 - 450,000/mm^3^
Fibrinogen	1,050mg/dL	200 - 400mg/dL
INR	1.1	0.9 - 1.3
CRP	52mg/dL	< 3mg/dL
Creatinine	1mg/dL	0.5 - 1.1mg/dL

INR - International Normalized Ratio; CRP - C-reactive protein.

**Table 2:** Thromboelastometry analysis

ROTEM	EXTEM	FIBTEM
CT (s)	80	77
CFT (s)	50	58
A 5 (mm)	33	29
A 10 (mm)	40	33
MCF (mm)	50	40
ML (%)	0	0

ROTEM - Rotational thromboelastometry; EXTEM - extrinsic rotational thromboelastometry; FIBTEM - Fibrinogen rotational thromboelastometry - clot firmness; CFT - clot formation time; A5 - amplitude 5; A10 - amplitude 10; MCF - maximum clot firmness; ML - maximum lysis.

## DISCUSSION

We aim to discuss the routine practice of platelet transfusion. This case report is an example of how prophylactic transfusion can be avoided using viscoelastic tests. We discuss a patient with severe thrombocytopenia associated with bone marrow aplasia who presented with septic shock and acute respiratory failure requiring mechanical ventilation. Bronchoscopy and bronchoalveolar lavage were requested for diagnosis. Thromboelastometry was performed to guide transfusion, and therapy was normal. This case illustrates the compensatory effect of increased fibrinogen as an acute phase reactant during sepsis on blood coagulability, even in patients with severe thrombocytopenia.

Thromboelastometry is a useful test for diagnosing blood disorders and for managing bleeding in critical care patients. In several cohort studies, bleeding management guided by thromboelastometry has been associated with a reduction in transfusion requirements and also a reduction in the incidence of transfusion-related adverse events, with a better patient outcome.^(9-11)^

Thrombocytopenia is not ideal for patients with bone marrow aplasia, which increases their exposure to blood products. As shown, the isolated platelet count is a poor predictor for bleeding risk. Nevertheless, the prophylactic use of platelet concentrates is quite common in many centers to prevent bleeding in patients undergoing invasive procedures or surgery.^(12)^

Massion et al. proposed that the increased fibrinogen concentration in septic patients may explain the discrepancy between low platelet levels and normal maximum clot firmness in thromboelastometry by compensating for thrombocytopenia or for decreased coagulation factor activity.^(13,14)^

Fibrinogen (factor I) is a soluble glycoprotein synthesized in the liver that plays a central role in the clot formation and stabilization process. It acts as the precursor of fibrin that gives a substrate to blood clots and also promotes platelet aggregation and fibrinolysis. The increased levels of fibrinogen in the blood lead to enhancement in platelet interaction due to increased binding to the platelet glycoprotein IIb/IIIa receptor and fibrinolysis impairment. Fibrinogen is an acute phase plasma protein whose synthesis and circulating concentration are upregulated in response to inflammation, infection and tissue injury, such that its blood concentration may increase up to ten-fold, enhancing thrombus formation by altering the kinetics of coagulation.

Clot strength as assessed by the MCF, a parameter of rotational thromboelastometry, is highly influenced by both fibrinogen levels and platelet count.^(7,8)^ The minimal platelet count for normal clot formation on viscoelastic tests is strongly affected by the fibrinogen level. Other than the platelet count, the MCF was the most important parameter in predicting bleeding in patients with idiopathic thrombocytopenic purpura.^(15)^

Therefore, patients with severe thrombocytopenia as we report in this case could benefit from thromboelastometry assessment in order to predict bleeding and avoid unnecessary transfusion, since platelets alone are not a good predictor of bleeding.

## CONCLUSION

Thromboelastometry used as a diagnostic tool for a clotting disorder prevented unnecessary prophylactic platelet transfusion considering the compensatory effect of increased fibrinogen concentration by sepsis, even in a patient with extremely severe thrombocytopenia. We believe that thromboelastometry may be a safer and more effective option in predicting the bleeding risk than isolated platelet counts in critically ill patients.
